# *In vivo *expression of the HBZ gene of HTLV-1 correlates with proviral load, inflammatory markers and disease severity in HTLV-1 associated myelopathy/tropical spastic paraparesis (HAM/TSP)

**DOI:** 10.1186/1742-4690-6-19

**Published:** 2009-02-19

**Authors:** Mineki Saito, Toshio Matsuzaki, Yorifumi Satou, Jun-ichirou Yasunaga, Kousuke Saito, Kimiyoshi Arimura, Masao Matsuoka, Yoshiro Ohara

**Affiliations:** 1 Department of Microbiology, Kanazawa Medical University, Ishikawa 920-0293, Japan; 2 Department of Neurology and Geriatrics, Kagoshima University Graduate School of Medical and Dental Sciences, Kagoshima 890-8520, Japan; 3 Laboratory of Virus Immunology, Institute for Virus Research, Kyoto University, Kyoto 606-8507, Japan; 4 Department of Immunology, Graduate School of Medicine, University of the Ryukyus, Okinawa 903-0215, Japan

## Abstract

**Background:**

Recently, human T-cell leukemia virus type 1 (HTLV-1) basic leucine zipper factor (HBZ), encoded from a minus strand mRNA was discovered and was suggested to play an important role in adult T cell leukemia (ATL) development. However, there have been no reports on the role of HBZ in patients with HTLV-1 associated inflammatory diseases.

**Results:**

We quantified the HBZ and tax mRNA expression levels in peripheral blood from 56 HTLV-1-associated myelopathy/tropical spastic paraparesis (HAM/TSP) patients, 10 ATL patients, 38 healthy asymptomatic carriers (HCs) and 20 normal uninfected controls, as well as human leukemic T-cell lines and HTLV-1-infected T-cell lines, and the data were correlated with clinical parameters. The spliced HBZ gene was transcribed in all HTLV-1-infected individuals examined, whereas tax mRNA was not transcribed in significant numbers of subjects in the same groups. Although the amount of HBZ mRNA expression was highest in ATL, medium in HAM/TSP, and lowest in HCs, with statistical significance, neither tax nor the HBZ mRNA expression per HTLV-1-infected cell differed significantly between each clinical group. The HTLV-1 HBZ, but not tax mRNA load, positively correlated with disease severity and with neopterin concentration in the cerebrospinal fluid of HAM/TSP patients. Furthermore, HBZ mRNA expression per HTLV-1-infected cell was decreased after successful immunomodulatory treatment for HAM/TSP.

**Conclusion:**

These findings suggest that *in vivo *expression of HBZ plays a role in HAM/TSP pathogenesis.

## Background

Human T-cell lymphotropic virus type 1 (HTLV-1) is a replication-competent human retrovirus [1,2] which is associated with adult T-cell leukemia (ATL) [3,4] and with a slowly progressive neurological disorder HTLV-1-associated myelopathy/tropical spastic paraparesis (HAM/TSP) [5,6]. In HTLV-1 infection, approximately 5% develop ATL [7] and another 2%-3% develop chronic inflammatory diseases involving the central nervous system (HAM/TSP), the eyes [8], the lungs [9], the joints [10], or the skeletal muscles [11]; most infected individuals, however, remain healthy in their lifetime (healthy asymptomatic carriers: HCs). Although the factors that cause these different manifestations of HTLV-1 infection are not fully understood, previous population association studies suggested that both viral and host genetic factors influence the outcome of infection [12].

Among several HTLV-1 genes, a transcriptional activator Tax encoded in the pX region is thought to play a central role in immortalization, oncogenesis and inflammation through its pleiotropic activity [13]. In HAM/TSP patients, it has been reported that several cytokines, chemokines and matrix metalloproteinases transactivated by Tax protein such as tumor necrosis factor-α (TNF-α) [14], monocyte chemoattractant protein-1 (MCP-1) [15] and matrix metalloproteinase (MMP)-9 [16] are overexpressed in the infiltrating mononuclear cells in the patients' spinal cords. In addition, a previous report from the United States suggested that the level of HTLV-1 tax mRNA expression in HTLV-1-infected cells (mRNA/DNA ratio) was significantly higher in HAM/TSP patients than HCs, and this finding correlated with the HTLV-1 proviral load, Tax-specific CD8+ T cell frequency and disease severity of the patients [17]. A report from Japan also indicated that HTLV-1 tax mRNA expression was higher in HAM/TSP than HCs, although the mRNA/DNA ratio was similar between both groups [18]. These results suggest an important role of Tax in the induction of HAM/TSP.

It has been reported that among fresh leukemic cells isolated from ATL patients, about 60% of cases do not express the tax transcript [19]. In tax transgenic mouse models, the mice develop a wide range of tumors such as neurofibrosarcomas, mesenchymal tumors, and mammary adenomas, or even skeletal abnormalities including osteolytic bone metastases [20-27]; however, no leukemias or lymphomas were identified except in three models, which used respectively the granzyme B promoter [28], Lck proximal promoter [29] and Lck distal promoter [30]. These findings suggest that Tax is required for malignant transformation but not essential for the maintenance of leukemic cells *in vivo*. Recently, a novel basic leucine zipper protein encoded by the complementary strand of the HTLV-1 genome, named HTLV-1 basic leucine zipper factor (HBZ), was characterized [31]. HBZ is expressed in all ATL cells [32], promotes proliferation of T-lymphocytes in its RNA form [32], suppresses Tax-mediated transactivation through the 5' LTR [31,33], promotes CD4+ T-lymphocyte proliferation in transgenic mice [32], and enhances infectivity and persistence in HTLV-1-inoculated rabbits [34].

In this study, we investigated whether HTLV-1 HBZ mRNA expression is associated with clinical and laboratory markers reported in HAM/TSP patients, including HTLV-1 proviral load, neopterin concentration in cerebrospinal fluid (CSF), and motor disability score. In addition, to confirm the previous observations [17,18], we have also investigated the tax mRNA expression in ATL patients, HAM/TSP patients, and HCs by using the same technology but in a larger number of subjects.

## Methods

### Patients and cells

Human leukemic T-cell lines (Jurkat, MOLT-4, and CEM) and HTLV-1-infected T-cell lines (C5/MJ, SLB1, HUT102, MT-1, MT-2, and MT-4) were cultured in RPMI 1640 medium supplemented with 10% FCS. The diagnosis of HAM/TSP was done in accordance with World Health Organization criteria [35]. The diagnosis of ATL was made on the basis of clinical features, hematological characteristics, serum antibodies against HTLV-1 antigens, and detection of the HTLV-1 viral genome inserted into leukemia cells by Southern blot hybridization. All the PBMC samples used in this study were collected prior to treatment by a Histopaque-1077 (Sigma) density gradient centrifugation, washed and stored in liquid nitrogen until use. This research was approved by the institutional review boards of the authors' institutions, and informed consent was obtained from all individuals.

### Quantification of HTLV-1 proviral load, tax and HBZ mRNA expression, anti-HTLV-1 antibody titers and neopterin concentration in cerebrospinal fluid

RNA was extracted from PBMCs using RNeasy Mini Kit with on-column DNase digestion (QIAGEN, Tokyo, Japan) according to the manufacturer's instructions. Complementary DNA (cDNA) was synthesized using TaqMan Gold RT-PCR Kit (Applied Biosystems, Tokyo, Japan). For cDNA synthesis from extracted mRNA, 2 μg total RNA, 10 μl 10×TaqMan RT buffer, 22 μl MgCl_2 _(25 mM), 20 μl dNTPs mixture (at a final concentration of 500 μM each), 5 μl random hexamers (50 μM), 2 μl RNase inhibitor (20 U/μl), and 2.5 μl (50 U/μl) Moloney murine leukemia virus reverse transcriptase were added to a total volume of 100 μl. Samples were incubated at 25°C for 10 minutes and 48°C for 30 minutes, and reactions were stopped by heating to 95°C for 5 minutes. Genomic DNA was extracted from the frozen PBMCs by QIAamp blood kit (QIAGEN, Tokyo, Japan). We, then, carried out a real time quantitative PCR using ABI Prism 7900 HT Fast Real-Time PCR System (Applied Biosystems) to examine the HTLV-1 proviral load [36] and tax mRNA expression [17] in PBMCs or HTLV-1 infected cell lines as reported previously. The amount of the HTLV-1 proviral load was calculated using β-actin as an internal control through the following formula: copy number of HTLV-1 tax per cell = [(copy number of tax)/(copy number of β-actin/2)]. The sequences of primers for HTLV-1 provirus were as follows: 5'-CAA ACC GTC AAG CAC AGC TT-3' and 5'-TCT CCA AAC ACG TAG ACT GGG T-3', and the probe was 5'-TTC CCA GGG TTT GGA CAG AGT CTT CT-3'. HBZ mRNA expression levels were also quantified by real time quantitative PCR using the same method for tax mRNA [17]. Namely, serially diluted cDNA from HTLV-1 infected MT-2 cells was used for generating standard curves for the value of HTLV-1 tax or HBZ mRNA and hypoxanthine ribosyl transferase (HPRT) mRNA, and the relative HTLV-1 tax or HBZ mRNA load was calculated by the following formula: HTLV-1 tax mRNA load = value of tax/value of HPRT. HTLV-1 HBZ mRNA load = value of HBZ/value of HPRT. We used aliquots of the same standard MT-2 cDNA preparation for all assays and the correlation values of standard curves were always more than 99%. The sequences of primers for tax mRNA detection were as follows: 5'-ATC CCG TGG AGA CTC CTC AA-3' and 5'-ATC CCG TGG AGA CTC CTC AA-3', and the probe was 5'-TCC AAC ACC ATG GCC CAC TTC CC-3'. The sequences of primers for HBZ mRNA detection were as follows: 5'-AGA ACG CGA CTC AAC CGG-3' and 5'-TGA CAC AGG CAA GCA TCG A-3', and the probe was 5'-TGG ATG GCG GCC TCA GGG CT-3'. As the probes for tax and HBZ mRNA surrounded the splice junction site of each mRNA, we detected HBZ splicing isoform, which is the most abundant HBZ transcript and contributed significantly to HBZ protein synthesis [37-39], but not unspliced form in this study. We used the HPRT primers and probe set (Applied Biosystems) for internal calibration. The tax and HBZ probes were labeled with fluorescent 6-carboxyfluorescein (FAM) (reporter) at the 5' end and fluorescent 6-carboxy tetramethyl rhodamine (TAMRA) (quencher) at the 3' end. All assays were performed in triplicate. The sensitivity of our real-time RT-PCR assay was determined using MT-2 cells diluted serially with PBMCs from a healthy uninfected donor. The HTLV-1 mRNA signal (both tax and HBZ) could be detected in a dose-dependent manner with a sensitivity limit as low as one MT-2 cell in 10^6 ^PBMCs. Neopterin levels were evaluated by HPLC with fluorometric detection methods as described previously [40]. Serum HTLV-1 antibody titers were determined by a particle agglutination method (Serodia-HTLV-1^®^, Fujirebio, Japan).

### Clinical evaluation

Motor dysfunction seen in HAM/TSP patients was evaluated by clinical neurologists according to the Osame Motor Disability Score (OMDS) [41], which grades motor dysfunction from zero (normal walking and running) to 13 (complete bedridden) as follows: 1 = normal gait but runs slow; 2 = abnormal gait; 3 = abnormal gait and unable to run; 4 = need support while using stairs; 5 = need one hand support in walking; 6 = need two hands support in walking; 7 = need two hands support in walking but is limited to 10 m; 8 = need two hands support in walking but is limited to 5 m; 9 = unable to walk but able to crawl on hands and knees; 10 = crawls with hands; 11 = unable to crawl but can turn sideways in bed; 12 = unable to turn sideways but can move the toes. We have used OMDS throughout our previous studies [41-43] because this is a neurological measure of disability weighted toward ambulation and was specifically developed to evaluate motor dysfunction seen in HAM/TSP patients. It is therefore more suitable for evaluating HAM/TSP motor symptoms than the widely used EDSS [44]. The laboratory data were examined by an investigator who was not involved in the patients' clinical care, and the neurologists who made the clinical evaluation did not have access to the laboratory data.

### Statistical analysis

The Mann-Whitney U test was used to compare data between two groups. Correlations between variables were examined by Spearman rank correlation analysis. Values of p < 0.05 were considered statistically significant.

## Results

### HTLV-1 tax and HBZ mRNA load in HAM/TSP, ATL and HCs

A total of 56 HAM/TSP patients, 10 ATL patients and 38 HCs completed the evaluation. Twenty normal uninfected healthy controls (NCs) were used as negative controls. The HTLV-1 proviral load in this study represents the copy number of HTLV-1 tax per cell (for HTLV-1 infected cell lines) or PBMC (for HAM/TSP, ATL and HCs) (Table 1). Therefore, the HTLV-1 proviral load represents the population of infected cells in PBMCs when one cell harbors one provirus. However, since recent data by Kamihira et al. indicated that 43 out of 321 ATL specimens (17.8%) showed two or more bands by Southern blot analysis after *EcoRI *digestion [45], we reviewed the Southern blot data of our 10 ATL patients. As a result, two distinct bands of over 9 kb were observed in *EcoRI *digestion in samples from two ATL patients, indicating at least the biclonal integration of HTLV-1 proviral DNA. The incidence of multibands in our cases (two out of ten: 20%) was comparable with the data by Kamihira et al. (17.8%). The number of HTLV-1 proviral load in MT-2 cells measured by our quantitative PCR method (16.2 copies/cell) was also comparable with the previous report (12.6 copies/cell) [46].

**Table 1:** HTLV-1 mRNA load, proviral load and mRNA/DNA ratio in HTLV-1 – infected individuals and T-cell lines.

Cell line	HBZ mRNA^a^	tax mRNA^b^	Proviral load^c^	HBZ mRNA/DNA^d^	tax mRNA/DNA^e^
C5/MJ	13.3	0.062	8.1	1.64	0.0076
HUT102	1.2	26.35	19.3	0.063	1.37
MT1	25.2	0.011	7.1	3.56	0.0015
MT2	7.8	1.24	16.2	0.48	0.077
MT4	2.4	1.71	12.6	0.19	0.135
SLB1	25.8	87.4	115.5	0.22	0.756

HAM/TSP*	0.74 (0.023–33.50)	0 (0–0.041)	0.051 (0.0008–0.41)	19.10 (0.81–273.45)	0 (0–0.32)
HCs*	0.15 (0.0013–6.42)	0 (0–0.000078)	0.0089 (0.0001–0.10)	16.67 (0.21–7358.91)	0 (0–0.11)
ATL*	31.43 (5.93–225.64)	0.000018 (0–0.59)	1.14 (0.25–2.88)	24.04 (13.77–135.83)	0 (0–0.29)

*The results represent the median and range (n = 56 for HAM/TSP, n = 38 for HCs and n = 10 for ATL)

^a^HTLV-1 HBZ mRNA load = value of HBZ/value of HPRT

^b^HTLV-1 tax mRNA load = value of tax/value of HPRT

^c^Proviral load: HTLV-1 tax copy number per cell

^d^HBZ mRNA/DNA ratio = HTLV-1 HBZ mRNA load/Proviral load

^e^tax mRNA/DNA ratio = HTLV-1 tax mRNA load/Proviral load

**Figure 1:**
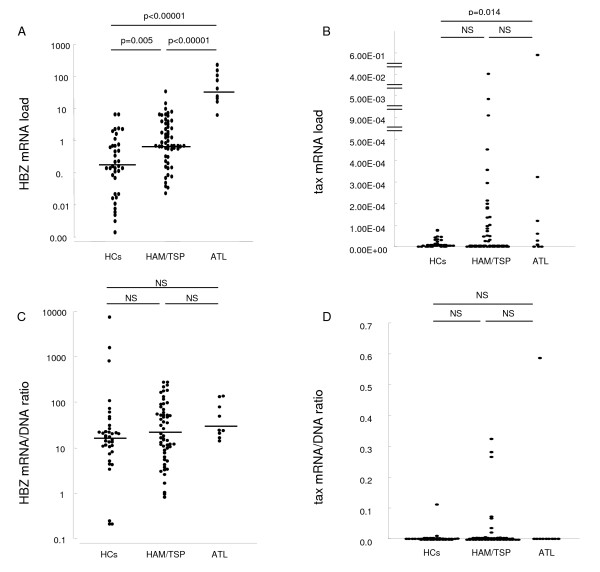
**HTLV-1 tax and HBZ mRNA load in patients with HAM/TSP, ATL and asymptomatic HTLV-I carriers**. A. HTLV-1 HBZ mRNA load was highest in ATL, medium in HAM/TSP, and lowest in HCs. B. The HTLV-1 tax mRNA load between HCs and HAM/TSP, HAM/TSP and ATL did not reach statistical significance, although the HTLV-1 tax mRNA load in ATL patients was significantly higher than HCs (p = 0.014, Mann Whitney U test). C and D. To normalize the HTLV-1 tax or HBZ mRNA expression level per provirus, the mRNA/DNA ratio was calculated by dividing the HTLV-1 tax or HBZ mRNA load by the HTLV-1 proviral load. Neither the HBZ (C) nor the tax (D) mRNA/DNA ratio differed significantly between each clinical group (HAM/TSP – HCs, HAM/TSP – ATL, HCs – ATL). The zero value of tax gene transcripts was observed in 60.7% of HAM/TSP patients (34 out of 56), 71.1% of HCs (27 out of 38) and 30.0% of ATL patients (3 out of 10). The medians are represented by horizontal lines and the statistical differences between them were calculated with a Mann Whitney U test.

The HTLV-1 proviral load was significantly greater in HAM/TSP patients (median 0.051, range 0.0008–0.41) than HCs (median 0.0089, range 0.0001–0.10) (P = 0.000011, Mann Whitney U test, Table 1). The HTLV-1 HBZ mRNA level was highest in ATL, medium in HAM/TSP, and lowest in HCs with statistical significance (Table 1 and Figure 1A). It is noteworthy that we could detect HTLV-1 HBZ gene transcripts in all infected individuals tested. Interestingly, there were three cases with extremely high data of HBZ mRNA in HCs (Figure 1C). Since recent report by Shimizu et al. indicated that HTLV-1-specific T-cell responsiveness widely differed among HTLV-1 carriers [47], these extremely high data of HBZ mRNA might be explained by immunological diversity observed in HCs. In contrast, although the HTLV-1 tax mRNA levels in ATL patients was significantly higher than HCs (p = 0.014, Mann-Whitney U test), the HTLV-1 tax mRNA levels between HCs-HAM/TSP and HAM/TSP-ATL did not reach statistical difference (Figure 1B). We could not detect any HTLV-1 tax and HBZ mRNA expression in any of the 20 NCs and 3 uninfected human leukemic T-cell lines (Jurkat, MOLT-4, and CEM) tested (data not shown).

### Comparison of HTLV-1 tax and HBZ mRNA load with HTLV-1 proviral load

**Figure 2:**
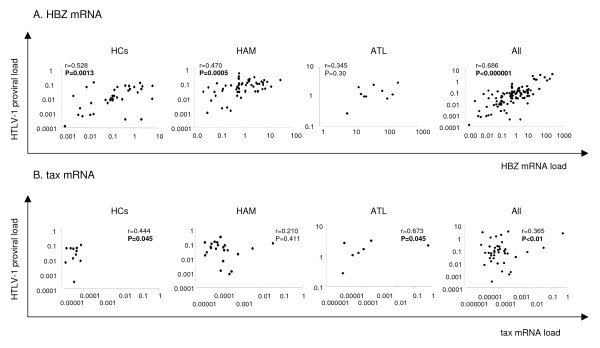
**Correlation between HTLV-1 proviral load and HTLV-1 mRNA load in HTLV-1 infected individuals**. A. The HTLV-1 HBZ mRNA load was significantly correlated with HTLV-1 proviral load in HAM/TSP patients alone (P = 0.0005, r = 0.470 by Spearman rank correlation analysis), HCs alone (P = 0.0013, r = 0.528) and all groups combined (P < 0.000001, r = 0.686) but not in ATL patients (P = 0.300, r = 0.345). B. The tax mRNA load correlated with the HTLV-1 proviral load in HCs (P = 0.045, r = 0.444), ATL patients (P = 0.045, r = 0.673) and both group combined (P < 0.01, r = 0.365) but not in HAM/TSP patients (P = 0.411, r = 0.210). The zero value of tax gene transcripts did not appear in the figures. Correlations were examined by Spearman rank correlation analysis.

To test whether higher HBZ mRNA levels reflect higher proviral load, we adjusted the tax or HBZ mRNA load (i.e. value of tax or HBZ/value of HPRT) by the HTLV-1 proviral load (i.e. HTLV-1 tax copy number per cell). As a result, neither tax nor the HBZ mRNA/DNA ratio differed significantly between each clinical group (i.e. HAM/TSP-HCs, HAM/TSP-ATL and HCs-ATL) (figure 1C, D). Interestingly, although both HTLV-1 proviral load and tax mRNA/DNA ratio were higher in HTLV-1-infected cell lines (C5/MJ, SLB1, HUT102, MT-1, MT-2, and MT-4) than PBMCs, HBZ mRNA/DNA ratio was even higher in PBMCs than HTLV-1-infected cell lines (Table 1). Consistent with the previous observations that HBZ suppresses Tax mediated transactivation through the 5' LTR [31,33,48], HBZ mRNA load tended to be higher in cell lines with lower tax mRNA load, and indeed HBZ mRNA/DNA ratio was inversely correlated with tax mRNA/DNA ratio in 6 HTLV-1-infected cell lines (Spearman's rank correlation coefficient r = -0.943, P = 0.035) (Table 1 and data not shown), although such correlation was not observed between HBZ and tax mRNA/DNA ratio in PBMCs from HAM/TSP patients, ATL patients, HCs and all groups combined (data not shown). As shown in Figure 2, the HTLV-1 HBZ mRNA load was significantly correlated with HTLV-1 proviral load in HAM/TSP patients (P = 0.0005, r = 0.470 by Spearman rank correlation analysis), HCs (P = 0.0013, r = 0.528) and all groups combined (P < 0.000001, r = 0.686), but not in ATL patients (P = 0.300, r = 0.345). The tax mRNA load was correlated with the HTLV-1 proviral load in HCs (P = 0.045, r = 0.444), ATL patients (P = 0.045, r = 0.673), and all groups combined (P < 0.01, r = 0.365), but not in HAM/TSP patients (P = 0.411, r = 0.210).

### Comparison of HBZ mRNA load with tax mRNA load among HTLV-1 infected individuals in different clinical status

**Figure 3:**
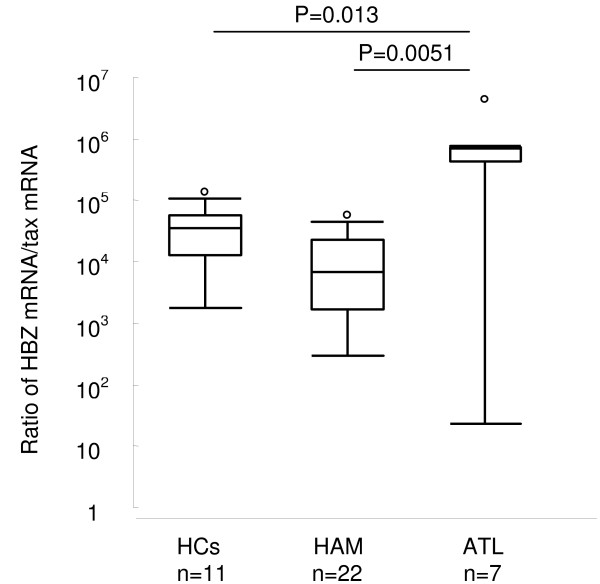
**Comparison of HBZ mRNA load with tax mRNA load among HTLV-1 infected individuals in different clinical status**. The ratio of HBZ mRNA/tax mRNA was significantly increased in ATL patients (median 700,512.24, range 23.11 – 4,308,413.02) than HAM/TSP patients (median 4,932.41, range 295.63–56,082.14) or HCs (median 35,602.96, range 1,804.77–137,999.33). The statistical differences between groups were calculated with a Mann Whitney U test.

To investigate the mutual expression status of HBZ and tax mRNA in different clinical status, we calculated the ratio of HBZ mRNA/tax mRNA in 22 HAM/TSP patients, 11 HCs and 7 ATL patients, who express both tax and HBZ mRNA in PBMCs. HTLV-1 tax mRNA was not expressed in 60.7% (34 out of 56) of HAM/TSP patients, 71.1% (27 out of 38) of HCs and 30.0% (3 out of 10) of ATL patients, whereas HTLV-1 HBZ mRNA was expressed in all the infected individuals tested. As shown in figure 3, HBZ mRNA/tax mRNA ratio in PBMCs was significantly increased in ATL patients than HAM/TSP patients and HCs (P = 0.013 and 0.0051, Mann-Whitney U test, respectively), indicating very high HBZ transcript levels relative to tax, especially in ATL patients.

### Correlation of HTLV-1 HBZ mRNA load with CSF neopterin concentration and disease severity in HAM/TSP patients

To investigate the relationship between HTLV-1 mRNA load and various laboratory markers, HTLV-1 proviral load, CSF neopterin concentration and anti-HTLV-1 antibody titers were quantified and compared with motor dysfunction of HAM/TSP patients. Since neopterin is a low molecular weight pteridine compound released from macrophages upon stimulation with γ-interferon secreted by activated T cells, the measurement of neopterin concentrations in body fluids like blood serum, CSF or urine provides information about cellular immune activation in humans under the control of type 1 T helper cells [49]. As shown in table 2, we showed that the CSF neopterin level, which was positively correlated with proviral load, was also positively correlated with the HBZ mRNA load in HAM/TSP patients (Spearman's rank correlation coefficient P = 0.0052, r = 0.437). However, such a correlation was not observed between neopterin and HTLV-1 tax mRNA load (P = 0.544, r = 0.228). Motor dysfunction evaluated by OMDS significantly correlated with HTLV-1 HBZ mRNA load (P = 0.023, r = 0.328), but again not with HTLV-1 tax mRNA load (P = 0.401, r = 0.241).

**Table 2:** Results of rank correlation test between clinical and virological parameters.

	Proviral load		HBZ mRNA^a^		tax mRNA^b^		HBZ mRNA/DNA^c^		tax mRNA/DNA^d^	
	
	r	p	r	p	r	p	r	p	r	p
OMDS	0.169	0.285	0.328	**0.023**	0.241	0.401	0.252	0.091	0.257	0.300
Neopterin in CSF	0.512	**0.001**	0.437	**0.0052**	0.228	0.544	0.121	0.442	0.211	0.608
Serum Ab	0.117	0.431	0.185	0.194	0.234	0.333	0.102	0.497	0.248	0.279
CSF Ab	0.071	0.639	0.042	0.801	-0.0029	0.322	-0.046	0.690	0.0025	0.345

OMDS: Osame Motor Disability Scale for HAM/TSP

^a^HTLV-1 HBZ mRNA load = value of HBZ/value of HPRT

^b^HTLV-1 tax mRNA load = value of tax/value of HPRT

^c^HBZ mRNA/DNA ratio = HTLV-1 HBZ mRNA load/Proviral load

^d^tax mRNA/DNA ratio = HTLV-1 tax mRNA load/Proviral load

### HBZ mRNA load and HBZ mRNA/DNA ratio in PBMCs was decreased in HAM/TSP patients after effective IFN-α treatment

**Figure 4:**
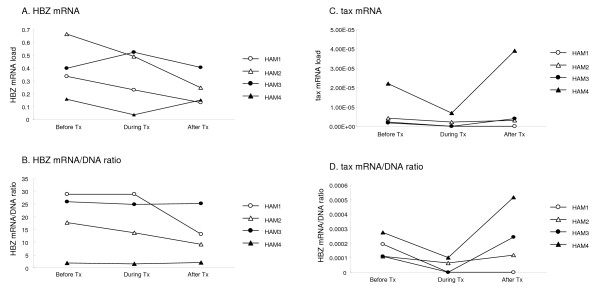
**HBZ mRNA load and HBZ mRNA/DNA ratio in PBMCs were decreased in HAM/TSP patients after effective IFN-α treatment**. To investigate whether HTLV-1 mRNA load and mRNA/DNA ratio are associated with clinical improvement, we measured the HBZ mRNA/DNA ratio in four HAM/TSP patients who received 4 weeks of daily IFN-α administration (three million international units of IFN-α per one intramuscular injection). Two HAM/TSP patients with clinical improvement in Osame Motor Disability Score (OMDS) (HAM1 and 2) showed decreased HBZ mRNA load and HBZ mRNA/DNA ratio during the IFN-α treatment, whereas two HAM/TSP patients without clinical improvement in OMDS (HAM3 and 4) showed stable HBZ mRNA load and HBZ mRNA/DNA ratio during the IFN-α treatment. In contrast, the tax mRNA load and tax mRNA/DNA ratio did not show such a clear correlation with clinical improvement.

Finally, to determine whether HTLV-1 mRNA load and mRNA/DNA ratio are associated with clinical improvement, we measured the HTLV-1 (both tax and HBZ) mRNA load and mRNA/DNA ratio before, during, and after interferon-alpha (IFN-α) treatment in four HAM/TSP patients who received 4 weeks of daily administration. Three million international units (IU) of IFN-α (human lymphoblastoid interferon-HLBI, Sumiferon^® ^by Sumitomo Pharmaceutical Co., Osaka, Japan) were administrated per intramuscular injection. Two patients (HAM1 and 2) showed marked clinical improvement with the changes of the OMDS, whereas two patients (HAM3 and 4) did not show clinical improvement (without the changes of the OMDS) (Additional file 1). The HBZ mRNA load and mRNA/DNA ratio was decreased after IFN-α treatment in two patients who showed clinical improvement, whereas the HBZ mRNA load and mRNA/DNA ratio was stable during the treatment in two patients without clinical improvement (Additional file 1 and Figure 4). In contrast, the tax mRNA load and mRNA/DNA ratio did not show such a clear correlation with clinical improvement.

## Discussion

In this study, we demonstrated that there was a statistically significant difference in the HTLV-1 HBZ mRNA load, but not tax mRNA load, in PBMCs between HAM/TSP patients and HCs. This is probably because tax mRNA was not expressed in significant numbers of individuals tested (60.7% of HAM/TSP patients, 34 out of 56; 71.1% of HCs, 27 out of 38; 30.0% of ATL patients, 3 out of 10), whereas HTLV-1 HBZ mRNA was expressed in all the infected individuals tested. There was also a statistically significant correlation between HTLV-1 HBZ mRNA load and HTLV-1 proviral load both in HAM/TSP patients and HCs, whereas tax mRNA load correlated with the HTLV-1 proviral load only in HCs but not in HAM/TSP patients. Recently, Usui et al. reported a similar observation [37]. Namely, HBZ spliced isoform mRNA was detectable in samples from most HCs and ATL patients, and was significantly correlated with the HTLV-1 proviral load. These results indicate that the regulation of HBZ mRNA expression is different from that of tax mRNA. It seems likely that HBZ mRNA is near-equally expressed by all provirus-positive cells despite different clinical status, while tax mRNA expression levels are variable in different clinical status.

When HTLV-1 tax or HBZ mRNA load was adjusted with HTLV-1 proviral DNA load (i.e. calculate mRNA/DNA ratio), the amount of tax and HBZ mRNA expressed per provirus was not significantly different between HAM/TSP patients and HCs, suggesting that the higher HTLV-1 proviral load seen in HAM/TSP patients caused higher HTLV-1 HBZ mRNA expression. This is consistent with our previous study using different methods for mRNA and DNA quantification [18], but differed from a previous American study using exactly the same methods, which showed significantly higher mRNA/DNA ratio in HAM/TSP patients than HCs [17]. In contrast to the previous study, which showed significant correlation between disease severity in HAM/TSP patients and both HTLV-1 tax mRNA load and mRNA/DNA ratio [17], we could not find such a correlation between clinical parameters of HAM/TSP patients including disease severity and both HTLV-1 tax mRNA load and mRNA/DNA ratio (Table 2). As we have already confirmed and reported the same levels of Tax protein expression in HTLV-1-infected PBMCs between HAM/TSP patients and HCs in the same cohort [50], the observed discrepancy may be due to the differences of a number of host genetic and virologic factors in HTLV-1 infected individuals, including differences in HLA haplotypes [51-53], differences in the amount of soluble suppressive factors and CD8+ T-cell responses, and differences in HTLV-1 tax genomic sequences [54]. As a recent report indicated that HTLV-I infection was associated with activated T-cell immunity in Jamaicans but with diminished T-cell immunity in Japanese persons [55], the interaction between different genes and/or environmental factors is also likely to contribute to the observed differences between the two populations. Namely, genetic resistance to infectious diseases that is formed by complex host genetic effects might be complicated further by pathogen diversity and environmental factors.

Another important observation is that the amount of HTLV-1 HBZ mRNA expression per provirus was more than a thousand times higher than tax mRNA expression both in HAM/TSP patients and HCs. Surprisingly, the amount of HTLV-1 HBZ mRNA expression per provirus was even higher in HTLV-1-infected PBMCs than in infected cell lines, whereas tax mRNA expression was significantly higher in cell lines than infected PBMCs. Since HBZ suppresses Tax-mediated viral transcription [31], the abundant expression of HBZ mRNA in HTLV-1-infected PBMCs will be one of the molecular mechanisms involved in viral latency by suppressing HTLV-1 transcription and Tax expression, which may be a significant advantage to the virus in the infected cell by preventing its detection through a CTL response. Since we and others [37] found that down-regulation of tax mRNA (higher HBZ mRNA/tax mRNA ratio) was characteristic of primary ATL cells, imbalanced expression between HBZ and tax may induce the outgrowth of HTLV-1-transformed T cell and increase the risk of ATL, which is associated with a Tax-low or -negative phenotype.

We also found that the HTLV-1 HBZ mRNA load significantly correlated with the neopterin concentrations in CSF of HAM/TSP patients. Since neopterin levels in CSF have been used as an immunologic marker for monitoring disease activity and treatment efficacy of HAM/TSP [40,42,56], the quantitative analysis of HTLV-1 HBZ mRNA might also be used to monitor HAM/TSP disease activity. As expected, motor dysfunction of HAM/TSP patients evaluated by the OMDS score significantly correlated with HTLV-1 HBZ mRNA load (P = 0.023) but not with HTLV-1 tax mRNA load (P = 0.401). The correlation between HBZ mRNA load and two independent clinical parameters reflecting disease activities strongly suggest its stronger relevance than both tax mRNA and proviral load for HAM/TSP pathogenesis. This is further supported by the data that both HBZ mRNA load and HBZ mRNA/DNA ratio were decreased in HAM/TSP patients after effective IFN-α treatment. Collectively, our results suggest that higher HTLV-1 HBZ mRNA load may have relative prognostic value for the assessment of disease progression and could also be used as a surrogate marker to predict long-term outcome in HAM/TSP patients.

In summary, we showed that spliced HBZ gene was transcribed in all the HTLV-1 infected individuals examined, whereas tax mRNA was not transcribed in more than half in the same groups. Moreover, our data demonstrated a significant correlation between HTLV-1 HBZ mRNA load and HTLV-1 proviral load, neopterin concentrations in CSF and motor disability seen in HAM/TSP patients, indicating that HTLV-1 HBZ mRNA load may be a valid predictor of disease progression. Our present findings suggest that HTLV-1 HBZ mRNA expression plays a role not only in ATL, but also in the pathogenesis of the HTLV-1-associated inflammatory disease HAM/TSP.

## Competing interests

The authors declare that they have no competing interests.

## Authors' contributions

MS designed and performed the experiments, analyzed the data, and wrote the paper; TM and KA provided clinical samples and assembled clinical database. YS and JY provided clinical samples and performed experiments. KS performed experiments, analyzed and interpreted data. MM made contribution to the conception and design of the study. YO contributed to obtaining funding and gave advice.

## Supplementary Material

Additional file 1 Changes in HBZ mRNA load and HBZ mRNA/DNA ratio in PBMCs of HAM/TSP patients after IFN-α treatment. Click here for file

## References

[B1] Poiesz BJ, Ruscetti FW, Gazdar AF, Bunn PA, Minna JD, Gallo RC (1980). Detection and isolation of type C retrovirus particles from fresh and cultured lymphocytes of a patient with cutaneous T-cell lymphoma. Proc Natl Acad Sci USA.

[B2] Yoshida M, Miyoshi I, Hinuma Y (1982). Isolation and characterization of retrovirus from cell lines of human adult T-cell leukemia and its implication in the disease. Proc Natl Acad Sci USA.

[B3] Hinuma Y, Nagata K, Hanaoka M, Nakai M, Matsumoto T, Kinoshita KI, Shirakawa S, Miyoshi I (1981). Adult T-cell leukemia: antigen in an ATL cell line and detection of antibodies to the antigen in human sera. Proc Natl Acad Sci USA.

[B4] Yoshida M, Seiki M, Yamaguchi K, Takatsuki K (1984). Monoclonal integration of human T-cell leukemia provirus in all primary tumors of adult T-cell leukemia suggests causative role of human T-cell leukemia virus in the disease. Proc Natl Acad Sci USA.

[B5] Gessain A, Barin F, Vernant JC, Gout O, Maurs L, Calender A, de The G (1985). Antibodies to human T-lymphotropic virus type-I in patients with tropical spastic paraparesis. Lancet.

[B6] Osame M, Usuku K, Izumo S, Ijichi N, Amitani H, Igata A, Matsumoto M, Tara M (1986). HTLV-I associated myelopathy, a new clinical entity. Lancet.

[B7] Arisawa K, Soda M, Endo S, Kurokawa K, Katamine S, Shimokawa I, Koba T, Takahashi T, Saito H, Doi H, Shirahama S (2000). Evaluation of adult T-cell leukemia/lymphoma incidence and its impact on non-Hodgkin lymphoma incidence in southwestern Japan. Int J Cancer.

[B8] Mochizuki M, Watanabe T, Yamaguchi K, Takatsuki K, Yoshimura K, Shirao M, Nakashima S, Mori S, Araki S, Miyata N (1992). HTLV-I uveitis: a distinct clinical entity caused by HTLV-I. Jpn J Cancer Res.

[B9] Sugimoto M, Nakashima H, Watanabe S, Uyama E, Tanaka F, Ando M, Araki S, Kawasaki S (1987). T-lymphocyte alveolitis in HTLV-I-associated myelopathy. Lancet.

[B10] Nishioka K, Maruyama I, Sato K, Kitajima I, Nakajima Y, Osame M (1989). Chronic inflammatory arthropathy associated with HTLV-I. Lancet.

[B11] Higuchi I, Montemayor ES, Izumo S, Inose M, Osame M (1993). Immunohistochemical characteristics of polymyositis in patients with HTLV-I-associated myelopathy and HTLV-I carriers. Muscle Nerve.

[B12] Bangham CR, Osame M (2005). Cellular immune response to HTLV-1. Oncogene.

[B13] Yoshida M (2001). Multiple viral strategies of HTLV-1 for dysregulation of cell growth control. Annu Rev Immunol.

[B14] Umehara F, Izumo S, Ronquillo AT, Matsumuro K, Sato E, Osame M (1994). Cytokine expression in the spinal cord lesions in HTLV-I-associated myelopathy. J Neuropathol Exp Neurol.

[B15] Umehara F, Izumo S, Takeya M, Takahashi K, Sato E, Osame M (1996). Expression of adhesion molecules and monocyte chemoattractant protein -1 (MCP-1) in the spinal cord lesions in HTLV-I-associated myelopathy. Acta Neuropathol (Berl).

[B16] Umehara F, Okada Y, Fujimoto N, Abe M, Izumo S, Osame M (1998). Expression of matrix metalloproteinases and tissue inhibitors of metalloproteinases in HTLV-I-associated myelopathy. J Neuropathol Exp Neurol.

[B17] Yamano Y, Nagai M, Brennan M, Mora CA, Soldan SS, Tomaru U, Takenouchi N, Izumo S, Osame M, Jacobson S (2002). Correlation of human T-cell lymphotropic virus type 1 (HTLV-1) mRNA with proviral DNA load, virus-specific CD8(+) T cells, and disease severity in HTLV-1-associated myelopathy (HAM/TSP). Blood.

[B18] Furukawa Y, Osame M, Kubota R, Tara M, Yoshida M (1995). Human T-cell leukemia virus type-1 (HTLV-1) Tax is expressed at the same level in infected cells of HTLV-1-associated myelopathy or tropical spastic paraparesis patients as in asymptomatic carriers but at a lower level in adult T-cell leukemia cells. Blood.

[B19] Matsuoka M (2005). Human T-cell leukemia virus type I (HTLV-I) infection and the onset of adult T-cell leukemia (ATL). Retrovirology.

[B20] Hinrichs SH, Nerenberg M, Reynolds RK, Khoury G, Jay G (1987). A transgenic mouse model for human neurofibromatosis. Science.

[B21] Nerenberg M, Hinrichs SH, Reynolds RK, Khoury G, Jay G (1987). The tat gene of human T-lymphotropic virus type 1 induces mesenchymal tumors in transgenic mice. Science.

[B22] Green JE, Hinrichs SH, Vogel J, Jay G (1989). Exocrinopathy resembling Sjogren's syndrome in HTLV-1 tax transgenic mice. Nature.

[B23] Iwakura Y, Tosu M, Yoshida E, Takiguchi M, Sato K, Kitajima I, Nishioka K, Yamamoto K, Takeda T, Hatanaka M (1991). Induction of inflammatory arthropathy resembling rheumatoid arthritis in mice transgenic for HTLV-I. Science.

[B24] Ruddle NH, Li CB, Horne WC, Santiago P, Troiano N, Jay G, Horowitz M, Baron R (1993). Mice transgenic for HTLV-I LTR-tax exhibit tax expression in bone, skeletal alterations, and high bone turnover. Virology.

[B25] Hall AP, Irvine J, Blyth K, Cameron ER, Onions DE, Campbell ME (1998). Tumours derived from HTLV-I tax transgenic mice are characterized by enhanced levels of apoptosis and oncogene expression. J Pathol.

[B26] Gao L, Deng H, Zhao H, Hirbe A, Harding J, Ratner L, Weilbaecher K (2005). HTLV-1 Tax transgenic mice develop spontaneous osteolytic bone metastases prevented by osteoclast inhibition. Blood.

[B27] Furuta Y, Aizawa S, Suda Y, Ikawa Y, Kishimoto H, Asano Y, Tada T, Hikikoshi A, Yoshida M, Seiki M (1989). Thymic atrophy characteristic in transgenic mice that harbor pX genes of human T-cell leukemia virus type I. J Virol.

[B28] Grossman WJ, Kimata JT, Wong FH, Zutter M, Ley TJ, Ratner L (1995). Development of leukemia in mice transgenic for the tax gene of human T-cell leukemia virus type I. Proc Natl Acad Sci USA.

[B29] Hasegawa H, Sawa H, Lewis MJ, Orba Y, Sheehy N, Yamamoto Y, Ichinohe T, Tsunetsugu-Yokota Y, Katano H, Takahashi H, Matsuda J, Sata T, Kurata T, Nagashima K, Hall WW (2006). Thymus-derived leukemia-lymphoma in mice transgenic for the Tax gene of human T-lymphotropic virus type I. Nat Med.

[B30] Ohsugi T, Kumasaka T, Okada S, Urano T (2007). The Tax protein of HTLV-1 promotes oncogenesis in not only immature T cells but also mature T cells. Nat Med.

[B31] Gaudray G, Gachon F, Basbous J, Biard-Piechaczyk M, Devaux C, Mesnard JM (2002). The complementary strand of the human T-cell leukemia virus type 1 RNA genome encodes a bZIP transcription factor that down-regulates viral transcription. J Virol.

[B32] Satou Y, Yasunaga J, Yoshida M, Matsuoka M (2006). HTLV-I basic leucine zipper factor gene mRNA supports proliferation of adult T cell leukemia cells. Proc Natl Acad Sci USA.

[B33] Basbous J, Arpin C, Gaudray G, Piechaczyk M, Devaux C, Mesnard JM (2003). The HBZ factor of human T-cell leukemia virus type I dimerizes with transcription factors JunB and c-Jun and modulates their transcriptional activity. J Biol Chem.

[B34] Arnold J, Yamamoto B, Li M, Phipps AJ, Younis I, Lairmore MD, Green PL (2006). Enhancement of infectivity and persistence in vivo by HBZ, a natural antisense coded protein of HTLV-1. Blood.

[B35] Osame M (1990). Review of WHO Kagoshima meeting and diagnostic guidelines for HAM/TSP.

[B36] Nagai M, Usuku K, Matsumoto W, Kodama D, Takenouchi N, Moritoyo T, Hashiguchi S, Ichinose M, Bangham CR, Izumo S, Osame M (1998). Analysis of HTLV-I proviral load in 202 HAM/TSP patients and 243 asymptomatic HTLV-I carriers: high proviral load strongly predisposes to HAM/TSP. J Neurovirol.

[B37] Usui T, Yanagihara K, Tsukasaki K, Murata K, Hasegawa H, Yamada Y, Kamihira S (2008). Characteristic expression of HTLV-1 basic zipper factor (HBZ) transcripts in HTLV-1 provirus-positive cells. Retrovirology.

[B38] Murata K, Hayashibara T, Sugahara K, Uemura A, Yamaguchi T, Harasawa H, Hasegawa H, Tsuruda K, Okazaki T, Koji T, Miyanishi T, Yamada Y, Kamihira S (2006). A novel alternative splicing isoform of human T-cell leukemia virus type 1 bZIP factor (HBZ-SI) targets distinct subnuclear localization. J Virol.

[B39] Cavanagh MH, Landry S, Audet B, Arpin-Andre C, Hivin P, Pare ME, Thete J, Wattel E, Marriott SJ, Mesnard JM, Barbeau B (2006). HTLV-I antisense transcripts initiating in the 3'LTR are alternatively spliced and polyadenylated. Retrovirology.

[B40] Nomoto M, Utatsu Y, Soejima Y, Osame M (1991). Neopterin in cerebrospinal fluid: a useful marker for diagnosis of HTLV-I-associated myelopathy/tropical spastic paraparesis. Neurology.

[B41] Izumo S, Goto I, Itoyama Y, Okajima T, Watanabe S, Kuroda Y, Araki S, Mori M, Nagataki S, Matsukura S, Akamine T, Nakagawa M, Yamamoto I, Osame M (1996). Interferon-alpha is effective in HTLV-I-associated myelopathy: a multicenter, randomized, double-blind, controlled trial. Neurology.

[B42] Saito M, Nakagawa M, Kaseda S, Matsuzaki T, Jonosono M, Eiraku N, Kubota R, Takenouchi N, Nagai M, Furukawa Y, Usuku K, Izumo S, Osame M (2004). Decreased human T lymphotropic virus type I (HTLV-I) provirus load and alteration in T cell phenotype after interferon-alpha therapy for HTLV-I-associated myelopathy/tropical spastic paraparesis. J Infect Dis.

[B43] Matsuzaki T, Saito M, Usuku K, Nose H, Izumo S, Arimura K, Osame M (2005). A prospective uncontrolled trial of fermented milk drink containing viable Lactobacillus casei strain Shirota in the treatment of HTLV-1 associated myelopathy/tropical spastic paraparesis. J Neurol Sci.

[B44] Kurtzke JF (1983). Rating neurologic impairment in multiple sclerosis: an expanded disability status scale (EDSS). Neurology.

[B45] Kamihira S, Sugahara K, Tsuruda K, Minami S, Uemura A, Akamatsu N, Nagai H, Murata K, Hasegawa H, Hirakata Y, Takasaki Y, Tsukasaki K, Yamada Y (2005). Proviral status of HTLV-1 integrated into the host genomic DNA of adult T-cell leukemia cells. Clin Lab Haematol.

[B46] Hasegawa A, Ohashi T, Hanabuchi S, Kato H, Takemura F, Masuda T, Kannagi M (2003). Expansion of human T-cell leukemia virus type 1 (HTLV-1) reservoir in orally infected rats: inverse correlation with HTLV-1-specific cellular immune response. J Virol.

[B47] Shimizu Y, Takamori A, Utsunomiya A, Kurimura M, Yamano Y, Hishizawa M, Hasegawa A, Kondo F, Kurihara K, Harashima N, Watanabe T, Okamura J, Masuda T, Kannagi M (2008). Impaired Tax-specific T-cell responses with insufficient control of HTLV-1 in a subgroup of individuals at asymptomatic and smoldering stages. Cancer Sci.

[B48] Lemasson I, Lewis MR, Polakowski N, Hivin P, Cavanagh MH, Thebault S, Barbeau B, Nyborg JK, Mesnard JM (2007). Human T-cell leukemia virus type 1 (HTLV-1) bZIP protein interacts with the cellular transcription factor CREB to inhibit HTLV-1 transcription. J Virol.

[B49] Fuchs D, Hausen A, Reibnegger G, Werner ER, Dierich MP, Wachter H (1988). Neopterin as a marker for activated cell-mediated immunity: application in HIV infection. Immunol Today.

[B50] Furukawa Y, Kubota R, Eiraku N, Nakagawa M, Usuku K, Izumo S, Osame M (2003). Human T-cell lymphotropic virus type I (HTLV-I)-related clinical and laboratory findings for HTLV-I-infected blood donors. J Acquir Immune Defic Syndr.

[B51] Jeffery KJ, Usuku K, Hall SE, Matsumoto W, Taylor GP, Procter J, Bunce M, Ogg GS, Welsh KI, Weber JN, Lloyd AL, Nowak MA, Nagai M, Kodama D, Izumo S, Osame M, Bangham CR (1999). HLA alleles determine human T-lymphotropic virus-I (HTLV-I) proviral load and the risk of HTLV-I-associated myelopathy. Proc Natl Acad Sci USA.

[B52] Jeffery KJ, Siddiqui AA, Bunce M, Lloyd AL, Vine AM, Witkover AD, Izumo S, Usuku K, Welsh KI, Osame M, Bangham CR (2000). The influence of HLA class I alleles and heterozygosity on the outcome of human T cell lymphotropic virus type I infection. J Immunol.

[B53] Sabouri AH, Saito M, Usuku K, Bajestan SN, Mahmoudi M, Forughipour M, Sabouri Z, Abbaspour Z, Goharjoo ME, Khayami E, Hasani A, Izumo S, Arimura K, Farid R, Osame M (2005). Differences in viral and host genetic risk factors for development of human T-cell lymphotropic virus type 1 (HTLV-1)-associated myelopathy/tropical spastic paraparesis between Iranian and Japanese HTLV-1-infected individuals. J Gen Virol.

[B54] Furukawa Y, Yamashita M, Usuku K, Izumo S, Nakagawa M, Osame M (2000). Phylogenetic subgroups of human T cell lymphotropic virus (HTLV) type I in the tax gene and their association with different risks for HTLV-I-associated myelopathy/tropical spastic paraparesis. J Infect Dis.

[B55] Birmann BM, Breen EC, Stuver S, Cranston B, Martinez-Maza O, Falk KI, Okayama A, Hanchard B, Mueller N, Hisada M (2008). Population differences in immune marker profiles associated with human T-lymphotropic virus type I infection in Japan and Jamaica. Int J Cancer.

[B56] Nakagawa M, Nakahara K, Maruyama Y, Kawabata M, Higuchi I, Kubota H, Izumo S, Arimura K, Osame M (1996). Therapeutic trials in 200 patients with HTLV-I-associated myelopathy/tropical spastic paraparesis. J Neurovirol.

