# Supplementary material for: In vivo expression of the HBZ gene of HTLV-1 correlates with proviral load, inflammatory markers and disease severity in HTLV-1 associated myelopathy/tropical spastic paraparesis (HAM/TSP)
Source: Retrovirology. 2009 Feb 19;6:19. doi: 10.1186/1742-4690-6-19 (PMC2653460; doi:10.1186/1742-4690-6-19)
Supplement: Additional file 1 — Changes in HBZ mRNA load and HBZ mRNA/DNA ratio in PBMCs of HAM/TSP patients after IFN-α treatment. [file 1742-4690-6-19-S1.doc]

**Table S1: Changes in HBZ mRNA load and HBZ mRNA/DNA ratio in PBMCs of HAM/TSP patients after IFN-α treatment**

| Case | Age | Sex | Disease  duration (years) | Serum Ab* | CSF Ab | OMDS** | OMDS | tax mRNA ^a^ | | | tax mRNA/DNA ^b^ | | | HBZ mRNA ^c^ | | | HBZ mRNA/DNA ^d^ | | |
| --- | --- | --- | --- | --- | --- | --- | --- | --- | --- | --- | --- | --- | --- | --- | --- | --- | --- | --- | --- |
|  |  |  |  |  |  | Before  Tx | After Tx |  | | |  |  |  |  | | |  |  |  |
|  |  |  |  |  |  |  |  | Before  Tx | during  Tx | after  Tx | before  Tx | during  Tx | after  Tx | before Tx | during Tx | after Tx | before Tx | during Tx | after Tx |
| HAM1 | 58 | M | 4 | ×8192 | ×16 | 7 | 6 | 0.0000022 | 0 | 0 | 0.00019 | 0 | 0 | 0.335 | 0.229 | 0.131 | 28.8 | 13.2 | 13.2 |
| HAM2 | 61 | F | 12 | ×8192 | ×128 | 5 | 4 | 0.0000042 | 0.0000022 | 0.0000032 | 0.00011 | 0.000063 | 0.00012 | 0.665 | 0.488 | 0.248 | 13.7 | 9.1 | 9.1 |
| HAM3 | 69 | M | 20 | ×2048 | ×8 | 2 | 2 | 0.0000017 | 0 | 0.00000039 | 0.00011 | 0 | 0.0024 | 0.399 | 0.521 | 0.404 | 24.8 | 25.1 | 25.1 |
| HAM4 | 49 | M | 13 | ×8192 | ×32 | 5 | 5 | 0.000022 | 0.0000067 | 0.000039 | 0.00027 | 0.0001 | 0.00052 | 0.157 | 0.035 | 0.151 | 1.54 | 1.99 | 1.99 |

*Anti-HTLV-1 antibodies were titrated by the particle agglutination method.

** OMDS: Osame Motor Disability Score

^a^ HTLV-1 tax mRNA load = value of tax / value of HPRT

^b^ tax mRNA/DNA ratio = HTLV-1 tax mRNA load / HTLV-1 tax copy number per cell

^c^ HTLV-1 HBZ mRNA load = value of HBZ / value of HPRT

^d^ HBZ mRNA/DNA ratio = HTLV-1 HBZ mRNA load / HTLV-1 tax copy number per cell
